# *EZH2* mutations in chronic myelomonocytic leukemia cluster with *ASXL1* mutations and their co-occurrence is prognostically detrimental

**DOI:** 10.1038/s41408-017-0045-4

**Published:** 2018-01-22

**Authors:** Mrinal M. Patnaik, Rangit Vallapureddy, Terra L. Lasho, Katherine P. Hoversten, Christy M. Finke, Rhett Ketterling, Curtis Hanson, Naseema Gangat, Ayalew Tefferi

**Affiliations:** 1 Division of Hematology, Department of Internal Medicine, Mayo Clinic, Rochester, MN USA; 2 Department of Laboratory Medicine and Pathology, Mayo Clinic, Rochester, MN USA

Chronic myelomonocytic leukemia (CMML) is an aggressive hematological malignancy characterized by sustained peripheral blood monocytosis and an inherent risk for leukemic blast transformation^1,2^. Patients with CMML have between 10–15 mutations per kilobase of coding DNA regions; with majority of these (>90%) involving epigenetic regulator genes (*TET2* 60%, *ASXL1* 40%), splicing machinery (*SRSF2* 40%), and cell signaling (oncogenic *RAS* pathway 30%)^3–5^. The polycomb group proteins play an important role in transcriptional repression by regulating chromatin modifications and consist of two canonical polycomb repressive complexes (PRC) PRC1 and PRC2^6^. PRC2, comprises EED (embryonic ectoderm development protein), EZH2 (enhancer of zeste homolog 2), SUZ12 (suppressor of zeste 12 protein homolog), and RBBP4/7 (retinoblastoma binding protein), is recruited to chromatin and results in the trimethylation of lysine 27 of the histone 3 mark (H3K27me3), a repressive mark silencing gene transcription. In addition, *ASXL1* (additional sex combs like 1) has been shown to associate with PRC2 and results in global reductions in H3K27me3, suggesting a regulatory role in the PRC2^7^.

*ASXL1* mutations (frameshift and nonsense) result in the truncation of the ASXL1 protein, are seen in ~40% of CMML patients, and independently and adversely impact overall survival (OS)^4,8^. *EZH2* mutations (chromosome 7q36.1) in CMML, unlike in epithelial malignancies and lymphoproliferative disorders, are loss-of-function mutations and are uncommon (<5%), with an indeterminate prognostic impact^4,9^. Given that the co-occurrence of these mutations have been documented in myeloid neoplasms and that theoretically these could further impact the repressive role of the PRC2, we examined a large and informative CMML data set to assess the prognostic impact of *ASXL1* and *EZH2* co-mutations in CMML^10^.

Patients with 2016 WHO (World Health Organization)-defined CMML were identified from the institutional database^1^. Al.(BM) biopsies and cytogenetic studies performed at diagnosis. A 29 gene panel next-generation sequencing assay was carried out on BM DNA specimens on all 277 patients obtained at diagnosis for the following genes: *TET2*,* DNMT3A*,* IDH1*,* IDH2*, *ASXL1*,* EZH2*,* SUZ12*, *SRSF2*,* SF3B1*,* ZRSR2*,* U2AF1,* *PTPN11*,* Tp53*,* SH2B3*,* RUNX1*,* CBL*,* NRAS*,* KRAS*,* JAK2*,* CSF3R*,* FLT3*,* KIT*,* CALR*,* MPL,* *NPM1*,* CEBPA*,* IKZF*,* ETNK1*, and *SETBP1*, by previously described methods^5,9^. All statistical analyses considered parameters obtained at time of CMML diagnosis. Differences in the distribution of continuous variables between categories were analyzed by either Mann–Whitney or Kruskal–Wallis tests. Patient groups with nominal variables were compared by *χ*^2^ test. OS was calculated from the date of first referral to date of death or last contact. Leukemia-free survival (LFS) was calculated from the date of first referral to date of leukemic transformation or death/last contact. Overall and LFS curves were prepared by the Kaplan–Meier method and compared by the log-rank test. Cox proportional hazard regression model was used for multivariable analysis.

Two hundred and seventy-seven WHO-defined CMML patients were included in the study; median age 72 years (range, 18–92), 66% males. *ASXL1* mutations were identified in 138 (50%) patients, whereas *EZH2* mutations were identified in 7 (3%) patients; all 7 (100%) being co-mutated for *ASXL1* (Table 1). *EZH2* mutation types included; missense 3 (43%), nonsense 2 (28%), and 1 (14%) each for frameshift mutations and intronic mutations impacting splicing (splice site mutation). Four (57%) patients with *EZH2* mutations had a proliferative CMML phenotype, while three (43%) patients had an abnormal karyotype, including trisomy 8, isochromosome 17q, and a balanced translocation—*t*(3;12;6)(p21;q21;q23), respectively. Mutational frequencies in the *ASXL1/EZH2* co-mutated cohort included *TET2* 57%, *RUNX1* 43%, *SRSF2* 29%, and 14% each for *JAK2*V617F, *FLT3*-ITD, *BCOR, CBL, NRAS*, and *SETBP1*. Notably there were no mutations involving *DNMT3A*,* IDH1*,* IDH2*,* SF3B1*,* U2AF1*,* ZRSR2*, or *TP53*. In comparison to *ASXL1*mut/*EZH2*wt and *ASXL1*wt/*EZH2*wt, patients with *ASXL1/EZH2* co-mutations were more likely to have additional mutations involving *RUNX1* (43%, *p* = 0.001), *BCOR* (14%, *p* < 0.0001), *FLT3*-ITD (14%, *p* = 0.04) and less likely to have *SF3B1* mutations (0%, *p* = 0.003). The *ASXL1/EZH2* co-mutated patients were also more likely to have higher risk stratification by the *ASXL1* integrated Mayo Molecular Model (*p* < 0.0001) and the GFM CMML prognostic model (*p* < 0.0001).

**Table 1:** Clinical and laboratory characteristics of 277 CMML patients stratified by their *ASXL1* and *EZH2* mutational status

Variables	All patients with CMML (*n *= 277)	*ASXL1mt/EZH2mt* CMML patients (*n *= 7, 3%)	*ASXL1mt* CMML patients (*n *= 131, 47%)	*ASXL1wt* CMML patients (*n *= 139, 50%)	*P*-value comparing *ASXL1mt/EZH2mt* patients with *ASXL1m*t and *ASXL1wt*
Age in years; median (range)	72.3 (18–92)	67 (65–79)	72.5 (27–92)	72.4 (18–92)	0.7
Males; *n* (%)	183 (66)	6 (86)	89 (68)	88 (63)	0.4
Hemoglobin, g/dL; median (range)	10.7 (6.4–17)	11.3 (7.2–15)	10.7 (6.4–16.8)	10.9 (7.1–17)	0.7
WBC × 10^9^/L; median (range)	12.3 (2–265)	14.1 (6–51)	14.3 (2–265)	10 (2–186)	**0.01**
ANC × 10^9^/L; median (range)	6.2 (0.1–151	6.3 (3.2–32)	7.6 (0.2–151)	5.2 (0.1–143)	**0.04**
AMC × 10^9^/L; median (range)	2.5 (1–40)	2.8 (1–8)	3 (1–40)	2 (1–30)	**0.007**
ALC × 10^9^/L; median (range)	1.7 (0–22)	1.6 (1.2–6)	1.9 (0.4–22)	1.6 (0–11)	0.13
Platelets × 10^9^/L; median (range)	98 (10–840)	211 (25–526)	95 (10–726)	100 (12–840)	0.1
Presence of circulating immature myeloid cells; *n* (%)	156 (56)	5 (71)	81 (62)	70 (50)	0.09
PB blast %; median (range)	0 (0–19)	0 (0–4)	0 (0–19)	0 (0–12)	0.6
BM blast %; median (range)	3 (0–19)	1 (0–10)	4 (0–19)	3 (0–18)	0.08
Lactate dehydrogenase levels IU/ml; median (range)	226 (84–1296)	271 (163–604)	243 (84–1296)	215 (131–719)	0.1
Cytogenetics	(*n*=267)		(*n*=124)	(*n*=136)	0.7
abnormal; *n* (%)	85 (32)	3 (43)	41 (33)	41 (30)	
FAB CMML classification	(*n*=276)			(*n*=138)	**0.005**
Proliferative	135 (49)	4 (57)	77 (59)	54 (39)	
Dysplastic	141 (51)	3 (43)	54 (41)	84 (61)	
Therapy-related CMML; *n* (%)	28 (10	1 (14)	11 (8)	16 (12)	0.7
Next-generation sequencing analysis; *n* (%)					
1. Epigenetic regulators					
* TET2*	154 (56)	4 (57)	59 (45)	91 (65)	**0.003**
* DNMT3A*	16 (6)	0	7 (5)	9 (6)	0.7
* IDH1*	5 (2)	0	3 (2)	2 (1)	0.8
* IDH2*	16 (6)	0	8 (6)	8 (6)	0.8
2. Transcription factors					
* RUNX1*	21 (8)	3 (43)	11 (8)	7 (5)	**0.001**
* BCOR*	1 (0.5)	1 (14)	0	0	**<0.0001**
3. Spliceosome components					
* SF3B1*	15 (5)	0	1 (1)	14 (10)	**0.003**
* SRSF2*	129 (47)	2 (29)	63 (48)	64 (46)	0.6
* U2AF1*	19 (7)	0	12 (9)	7 (5)	0.3
* ZRSR2*	8 (3)	0	2 (2)	6 (4)	0.4
4. Cell signaling					
* JAK2 V617F*	20 (7)	1 (14)	9 (7)	10 (7)	0.8
* MPL*	2 (1)	0	0	2 (1)	0.4
* SH2B3*	1 (0.5)	0	1 (1)	0	0.6
* CBL*	37 (13)	0	20 (15)	17 (12)	0.4
* NRAS*	44 (16)	1 (14)	25 (19)	18 (13)	0.4
* KRAS*	12 (4)	1 (14)	8 (6)	3 (2)	0.1
* PTPN11*	8 (3)	0	5 (4)	3 (2)	0.6
* CSF3R*	4 (1)	0	2 (2)	2 (1)	0.9
* C-KIT*	8 (3)	0	4 (3)	4 (3)	0.9
* FLT3*	7 (3)	1 (14)	1 (1)	5 (4)	**0.04**
* NPM1*	1 (0.5)	0	0	1 (1)	0.6
* CALR*	1 (0.5)	0	0	1 (1)	0.6
5. Tumor suppressor genes					
* Tp53*	7 (3)	0	1 (1)	6 (4)	0.2
6. Others					
* SETBP1*	37 (13)	1 (14)	26 (20)	10 (7)	**0.009**
* CEBPA*	3 (1)	0	1 (1)	2 (1)	0.8
2016 WHO morphological subtypes; *n* (%)	(*n*=275)		(*n*=129)		0.9
CMML-0	155 (56)	5 (71)	73 (57)	77 (55)	
CMML-1	70 (25)	1 (14)	31 (24)	38 (27)	
CMML-2	50 (18)	1 (14)	25 (19)	24 (17)	
Spanish cytogenetic risk stratification; *n* (%)	(*n*=270)		(*n*=126)	(*n*=137)	0.5
Low	202 (75)	4 (57)	90 (71)	108 (79)	
Intermediate	42 (16)	2 (29)	22 (17)	18 (13)	
High	26 (10)	1 (14)	14 (11)	11 (18)	
Mayo-French cytogenetic risk stratification; *n* (%)	(*n*=270)		(*n*=126)	(*n*=137)	0.2
Low	201 (74)	4 (57)	89 (71)	108 (79)	
Intermediate	55 (20)	2 (29)	32 (25)	21 (15)	
High	14 (5)	1 (14)	5 (4)	8 (6)	
Mayo prognostic model; *n* (%)	(*n*=274)		(*n*=129)	(*n*=138)	0.14
Low	86 (31)	0	35 (27)	51 (37)	
Intermediate	88 (32)	4 (57)	45 (35)	39 (28)	
High	100 (36)	3 (43)	49 (38)	48 (35)	
Molecular Mayo model; *n* (%)	(*n*=260)		(*n*=126)	(*n*=127)	**<0.0001**
Low	20 (8)	0	0	20 (16)	
Intermediate-1	67 (26)	0	19 (15)	48 (38)	
Intermediate-2	83 (32)	4 (57)	37 (29)	42 (33)	
High	90 (35)	3 (43)	70 (56)	17 (13)	
GFM CMML prognostic model; *n* (%)	(*n*=268)		(*n*=129)	(*n*=132)	**<0.0001**
Low	117 (44)	1 (14)	29 (22)	87 (66)	
Intermediate	100 (37)	4 (57)	59 (46)	37 (28)	
High	51 (19)	2 (29)	41 (32)	8 (6)	
Leukemic transformation; *n* (%)	48 (17)	0	27 (21)	21 (15)	0.23
Deaths; *n* (%)	169 (61)	3 (43)	87 (66)	79 (57)	0.2
Follow-up in months; median (range)	16 (0.03–194)	6 (0.2–24)0.07-	15 (0.07–183)	19 (0.03–194)	0.08

The bold values represent statistically significant *p* values, *p* < 0.05

*mt* mutant, *wt* wild type, *WBC* white blood cell count, *ANC* absolute neutrophil count, *AMC* absolute monocyte count, *ALC* absolute lymphocyte count, *PB* peripheral blood, *BM* bone marrow, *WHO* World Health Organization, *GFM*Groupe Francophone des Myélodysplasies, *FAB*French–American–British, *BT* blast transformation

**Fig. 1:**
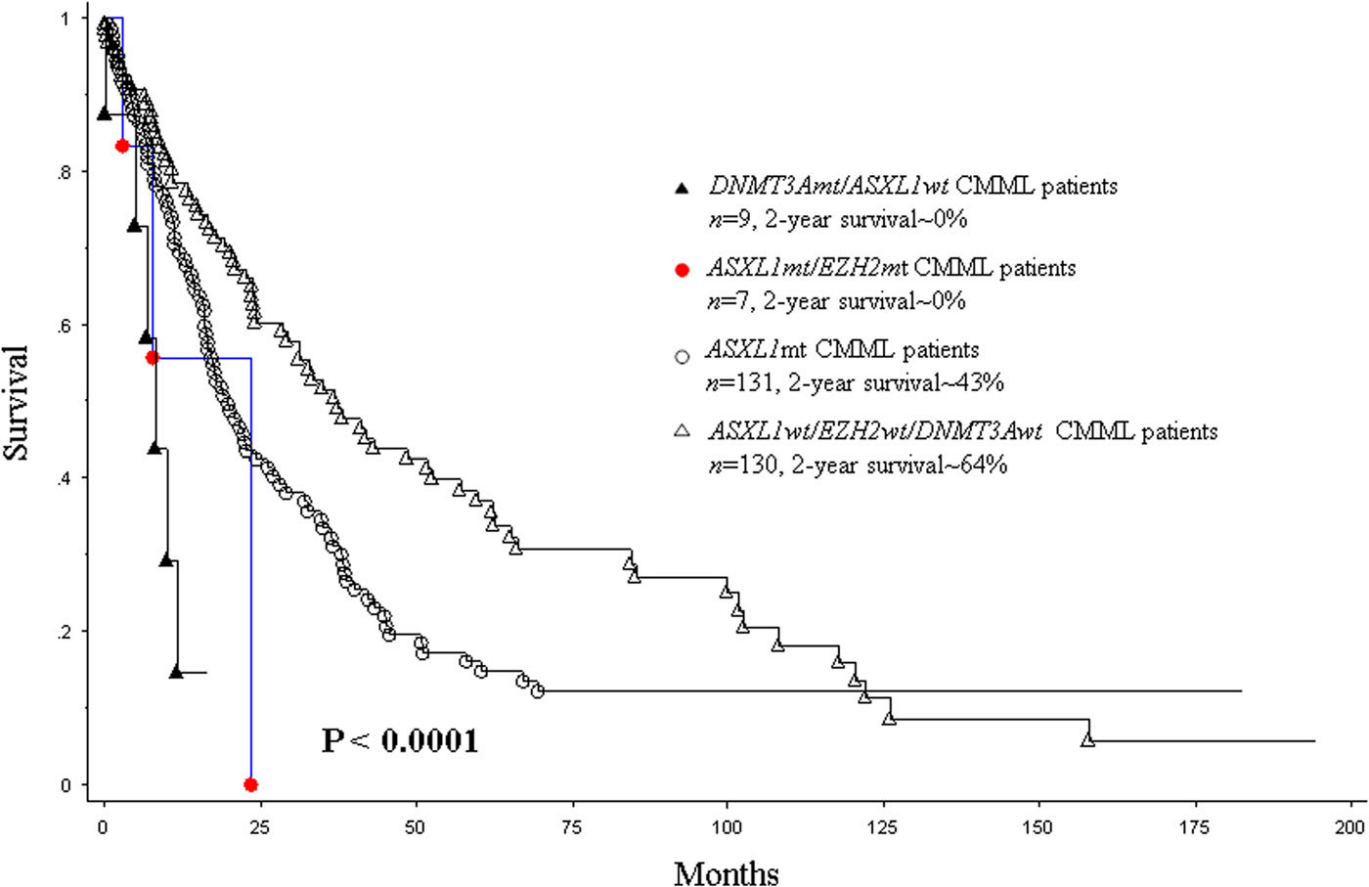
Overall survival of *ASXL1*, *EZH2*, and *DNMT3A* mutational status .

At last follow-up (median, 16 months), 169 (61%) deaths and 48 (17%) leukemic transformations were documented. Median survival for *ASXL1/EZH2* co-mutated patients was 16 months, in comparison to 20 months for *ASXL1*mt/*EZH2*wt and 33 months for *ASXL1*wt/*EZH2*wt patients (*p* < 0.0001, Fig. 1). In a univariate analysis, survival (OS) was adversely impacted by male sex (*p* = 0.03), low hemoglobin (HB < 10 gm/dl, *p* = 0.001), high white blood cell count (WBC > 15 × 10(9)/L; *p* = 0.0003), high absolute monocyte count (AMC > 10 × 10(9)/L, *p* = 0.0002), high absolute lymphocyte count (*p* = 0.02), presence of circulating immature myeloid cells (IMC, *p* = 0.002), peripheral blood (*p* = 0.001) and BM (*p* = 0.045) blast %, abnormal karyotype (*p* = 0.0008), absence of *TET2* mutations (*p* = 0.0003), presence of *ASXL1* (*p* = 0.009), *DNMT3A* (*p* = 0.001), and *Tp53* (*p* = 0.02) mutations. *EZH2* mutations by themselves did not impact OS (*p* = 0.2); however, when analyzed in the context of *ASXL1*mt/*EZH2*mt status, in comparison to *ASXL1*mutations, the adverse impact of the co-mutations was significantly stronger (*p* = 0.04, HR 2.9, 95% CI 1.1–9.5). In a multivariable survival analysis that included the aforementioned significant variables, only high WBC > 15 × 10(9)/L (*p* = 0.005, HR 1.005, 95% CI 1.003–1.009), male sex (*p* = 0.002, HR 1.7, 95% CI 1.2–2.4), presence of IMC (*p* = 0.009, HR 1.5, 95% CI 1.1–2.4), presence of *ASXL1/EZH2* co-mutations (*p* = 0.03, HR 2.1 95% CI 1.2–3.2), presence of *DNMT3A* mutations (*p* = 0.002, HR 2.8, 95% CI 1.4–5.4), and absence of *TET2* mutations (*p* = 0.0006, HR 1.7, 95% CI 1.2–2.4) independently and adversely impacted OS. The prognostic relevance of *ASXL1/EZH2* co-mutational status was lost when assessed in context of the Mayo Molecular Model (*p* = 0.2) and the GFM CMML model (*p* = 0.4). Neither did *ASXL1* (*p* = 0.14) nor *EZH2* (*p* = 0.3) mutations impact LFS.

In summary, our study has demonstrated that *EZH2* mutations are infrequent (<5%) in WHO-defined CMML, almost always co-occur with *ASXL1* mutations, are not associated with *DNMT3A* or *SF3B1* mutations, and are frequently associated with a “proliferative” CMML phenotype. While *EZH2* mutations themselves did not impact either OS or LFS, *ASXL1/EZH2* co-mutated patients had higher risk stratification by the *ASXL1*-integrated CMML prognostic models and had a shorter survival, in comparison to *ASXL1mt* patients alone. Mechanistic studies, including chromatin immunoprecipitation and sequencing (ChIP-seq) are needed to see if the *ASXL1/EZH2* co-mutational status indeed synergistically impacts PRC2 activity and further depletes methylation of H3K27, resulting in unbridled transcription.
